# Availability of published evidence on coverage, cost components, and funding support for digitalisation of infectious disease surveillance in Africa, 2003–2022: a systematic review

**DOI:** 10.1186/s12889-024-19205-2

**Published:** 2024-06-28

**Authors:** Basil Benduri Kaburi, Manuela Harries, Anja M. Hauri, Ernest Kenu, Kaspar Wyss, Bernard Chawo Silenou, Carolina J Klett-Tammen, Cordula Ressing, Jannis Awolin, Berit Lange, Gérard Krause

**Affiliations:** 1 Department of Epidemiology, Helmholtz Centre for Infection Research, Braunschweig, Germany; 2 PhD Programme “Epidemiology” Braunschweig-Hannover, Helmholtz Centre for Infection Research, Braunschweig, Germany; 3 Hannover Medical School, Hannover, Germany; 4 Ghana Field Epidemiology and Laboratory Training Programme, University of Ghana, Accra, Ghana; 5 Swiss Tropical and Public Health Institute, Allschwil, Switzerland; 6 University of Basel, Basel, Switzerland; 7 German Center for Infection Research partner site, Hannover-Braunschweig, Germany

**Keywords:** Implementation, Digital systems, Coverage, Infectious diseases, Surveillance, Outbreak response, Costing data, Funding, Sustainability, Africa

## Abstract

**Background:**

The implementation of digital disease surveillance systems at national levels in Africa have been challenged by many factors. These include user applicability, utility of IT features but also stable financial support. Funding closely intertwines with implementations in terms of geographical reach, disease focus, and sustainability. However, the practice of evidence sharing on geographical and disease coverage, costs, and funding sources for improving the implementation of these systems on the continent is unclear.

**Objectives:**

To analyse the key characteristics and availability of evidence for implementing digital infectious disease surveillance systems in Africa namely their disease focus, geographical reach, cost reporting, and external funding support.

**Methods:**

We conducted a systematic review of peer-reviewed and grey literature for the period 2003 to 2022 (PROSPERO registration number: CRD42022300849). We searched five databases (PubMed, MEDLINE over Ovid, EMBASE, Web of Science, and Google Scholar) and websites of WHO, Africa CDC, and public health institutes of African countries. We mapped the distribution of projects by country; identified reported implementation cost components; categorised the availability of data on cost components; and identified supporting funding institutions outside Africa.

**Results:**

A total of 29 reports from 2,033 search results were eligible for analysis. We identified 27 projects implemented in 13 countries, across 32 sites. Of these, 24 (75%) were pilot projects with a median duration of 16 months, (IQR: 5–40). Of the 27 projects, 5 (19%) were implemented for HIV/AIDs and tuberculosis, 4 (15%) for malaria, 4 (15%) for all notifiable diseases, and 4 (15%) for One Health. We identified 17 cost components across the 29 reports. Of these, 11 (38%) reported quantified costs for start-up capital, 10 (34%) for health personnel compensation, 9 (31%) for training and capacity building, 8 (28%) for software maintenance, and 7(24%) for surveillance data transmission. Of 65 counts of external funding sources, 35 (54%) were governmental agencies, 15 (23%) foundations, and 7 (11%) UN agencies.

**Conclusions:**

The evidence on costing data for the digitalisation of surveillance and outbreak response in the published literature is sparse in quantity, limited in detail, and without a standardised reporting format. Most initial direct project costs are substantially donor dependent, short lived, and thus unsustainable.

**Supplementary Information:**

The online version contains supplementary material available at 10.1186/s12889-024-19205-2.

## Background

The adoption of digital systems is increasingly recognised as essential for enhancing infectious disease surveillance and outbreak response [1]. The COVID-19 pandemic has shown the importance of digital systems for enhanced surveillance and outbreak response management at scale [2], and in real-time [3–5]. Beforehand, the occurrence of the West Africa Ebola outbreak, and the COVID-19 pandemic have accelerated the design and deployment of many digital tools to support response efforts [6–10].

Since 1998, countries of the World Health Organization Regional Office for Africa (WHO – AFRO) have adopted the Integrated Disease Surveillance and Response (IDSR) strategy [11]. It is a comprehensive, evidence-based strategy for strengthening national public health surveillance and response. It does so by integrating and harmonising the flow of surveillance data from community through to the national levels for early detection and response to public health threats [11]. With the occurrence of major public health emergencies in Africa, the limitations of the paper-based IDSR for early detection and coordination of emergency response became obvious [10, 12–17]. In 2013, with support of partners, the WHO initiated electronic surveillance termed “eSurveillance” to enhance the performance of the IDSR [18]. This enhancement constituted the use of electronic systems to facilitate rapid collection, early reporting, and analysis of both human and animal health data [11, 18]. In May 2023, the Africa CDC digital transformation strategy (2023–2030) was launched [19]. Among other commitments, the strategy undertakes to support the improvement of health systems capabilities of member countries to quickly detect, investigate and respond to health threats [19]. This strategy is aligned to Africa’s new public health order adopted by the African Union Commission in 2021 [20]. These efforts notwithstanding, there is currently no consolidated regional eSurveillance and outbreak response management system [21]. In the meantime, individual countries have adopted various digital systems to move forward with their respective IDSR.

The implementation of these digital systems at scale in African countries has been challenged by many factors – key among which are limited geographical reach and disease focus, and reliable financing [22, 23]. The levels of digitalisation and available funding in African public health systems are particularly low [24, 25]. Funding closely intertwines with implementations in terms of geographical reach, disease focus, and sustainability. The open sharing of evidence among African countries on their respective digitalisation experiences in respect of these factors could contribute to mitigating implementation failures. However, the practice of evidence sharing on geographical and disease coverage, costs, and funding sources for improving the implementation of these systems on the continent is unclear. Thus, even where there is stakeholder interest in appraising the long-term cost implications before undertaking a project, the required evidence or data may not be publicly available in the literature. This hinders realistic costing for successful piloting and scale-ups [26, 27]. The lack of a comprehensive appraisal and forecasting of the cost implications beyond the up-front costs contribute to implementation failures [28–30].

So, to assess the availability of published evidence for informing better planning and funding strategies for the digitalisation of surveillance and outbreak response in Africa, our review systematically addressed three thematic questions. First, what is the extent of geographical and disease coverage, and how long have the implemented surveillance systems been in operation? Second, how do published literature and reporting patterns illuminate project cost components, and what are the implications for strategic planning, piloting, and forecasting sustainable implementations at scale? Third, what are the sources of external funding support for African countries’ efforts to digitalise surveillance and outbreak response systems? Ultimately, the answers to these questions provide a gauge of the prevailing practices on documentation and transparency regarding implementation costs and funding sources.

## Methods

### Study design

We considered digital tools for public health surveillance and/or outbreak response to include smart phone or tablet-based approaches which are either SMS-, app- or web-based. We defined the limits of the review by geographical setting – Africa; public health conditions of interest – infectious diseases; purpose of project – surveillance and/or outbreak management, and the period of review – 2003 to 2022. We specified the components of our review question in terms of the “PICo” framework namely, the **P**opulation, **I**nterest, and **Co**ntext [31]. We developed a data extraction spreadsheet for recording relevant data from eligible records. We described and summarised the data in keeping with the review outcomes namely, geographical reach, disease focus, duration of implementation, cost components, and external funding support.

We registered the protocol for this systematic review in PROSPERO (CRD42022300849) (https://www.crd.york.ac.uk/PROSPERO/). We reported the review in keeping with the updated Preferred Reporting Items for Systematic Reviews and Meta-Analyses (PRISMA) guidelines 2020 [32].

### Literature search and study selection

#### Search strategy

We searched five electronic bibliographic databases and performed a manual search of cited references, websites of WHO, Africa CDC, and national institutes of public health of African countries. The electronic databases we searched were PubMed, MEDLINE over Ovid, EMBASE, Web of Science, and Google Scholar. We searched all fields for the period January 1, 2003 to December 31, 2022. We developed a stepwise search strategy using key words in our review starting in PubMed:

Search #1: cost OR cost components

Search #2: digital health tools

Search #3: infectious disease surveillance OR outbreak response

Search #4: Africa OR sub-Saharan Africa OR Low middle income country OR settings

Complete search (#5): #1 AND #2 AND #3 AND #4

Thus, we combined the four searches to obtain the complete set of search terms and results. With these keywords, PubMed generated additional related terms via its medical subject heading (MeSH) feature. Using Boolean operators and truncations, we adopted the search terms from PubMed for the other databases depending on the limits to number of search terms and unique search features [Appendix 1].

### Inclusion and exclusion criteria

The inclusion criteria hinged on three aspects: the project interests (digital applications involving infectious disease surveillance or outbreak response); location of projects (Africa); and type of publication (peer-reviewed articles, conference proceedings, and grey literature [33] on project reports on institutional websites, protocols). We excluded records on digital projects for non-communicable diseases, public health supply chain managements, health administration, and electronic medical records systems for routine patient care. By publication type, we excluded commentaries, opinion letters, and editorials.

### Study selection process

We imported all search results from electronic databases into EndNote X9 referencing system [34]. We performed duplicate detection and deletion. Next, we exported the remaining records onto the free web version of Rayyan for title and abstract screening [35]. The first author (BBK) and co-author (MH) performed a blinded title and abstract screening. We resolved 14 conflicts of screening decisions by consensus based on discussion. We obtained full texts of articles that passed the title and abstract screening. Three authors - BBK, MH, BCS independently performed eligibility assessment of full texts and agreed on included records.

### Quality assessment of reports

Based on the study designs of included records, we adapted appropriate items from the Critical Appraisal Skill’s Programme (CASP) check lists for economic evaluations [36], and the Appraisal tool for Cross-Sectional Studies (AXIS) [37] to obtain a 20 - item hybrid quality assessment tool (Appendix 2). The tool assessed the reports against six broad quality criteria namely, the clarity of research aims, appropriateness of methods, validity of results, discussion of implications of results, relevance of results for comparable settings, and compliance with ethics. It uses a four-level non-summative scoring system viz. “Yes”, “Can’t Tell”, “No”, and “Not Applicable” to assess each quality question.

### Data extraction process

We iteratively developed a data extraction spreadsheet in MS-Excel to capture a total of 45 variables in six broad themes. These themes were: title and source of report; purpose of project; features of implemented digital tools; sites of implementation, disease focus; duration of implementation, cost components as captured by reports; and names of funding institutions. Three authors - BBK, MH, and CR performed data extraction independently and subsequently merged results in one spreadsheet.

### Data management and analysis

In this review, we referred to results of our literature search as records. A record may contain two or more case studies. We referred to each case study as a report. Where a record reported separate case studies in different countries, we counted each country as different implementation site. Hence, the number of implementation sites was the total number of countries from which at least one project implementation was reported.

Geographic reach referred to government administrative divisions of a country such regions, districts, and sub-districts. The duration of implementation referred to the period for which a digital tool was deployed. We identified cost components as all implementation expenditure items or activity-based categories as they were captured in the reports. We listed all the cost components captured in included reports and organised them into activity and time-driven expenditure categories. We assessed the availability of data on cost components at four levels. For each report, a cost component was coded as “Unreported” if it was not reported; “Descriptive” if it was reported without any cost numbers; “Estimated” if it was reported with a cost estimate; “Quantified”, if it was reported and a full cost of expenditure provided. Where cost components were stated as free, such as the use of an open-source software, the cost component for software development was considered zero US dollars (USD), and hence categorised as “Quantified”.

External funding support referred to financial or technical support from countries or institutions other than the governments of beneficiary countries. For projects that received funding from multiple sources, we counted all funders for the tally. Where funds were in the form of a grant, we considered the main source of funding for the analysis.

We performed descriptive statistical analysis. The unit of analysis was a report. We tabulated key characteristics of the included reports, and the sources of external funding support. We visualised the data on geographical distribution of reporting countries, diseases for which the projects were implemented, durations of pilot projects, and the pattern of reporting on cost components across the four levels aforementioned.

## Results

### Literature search and evaluation for study inclusion

**Fig. 1:**
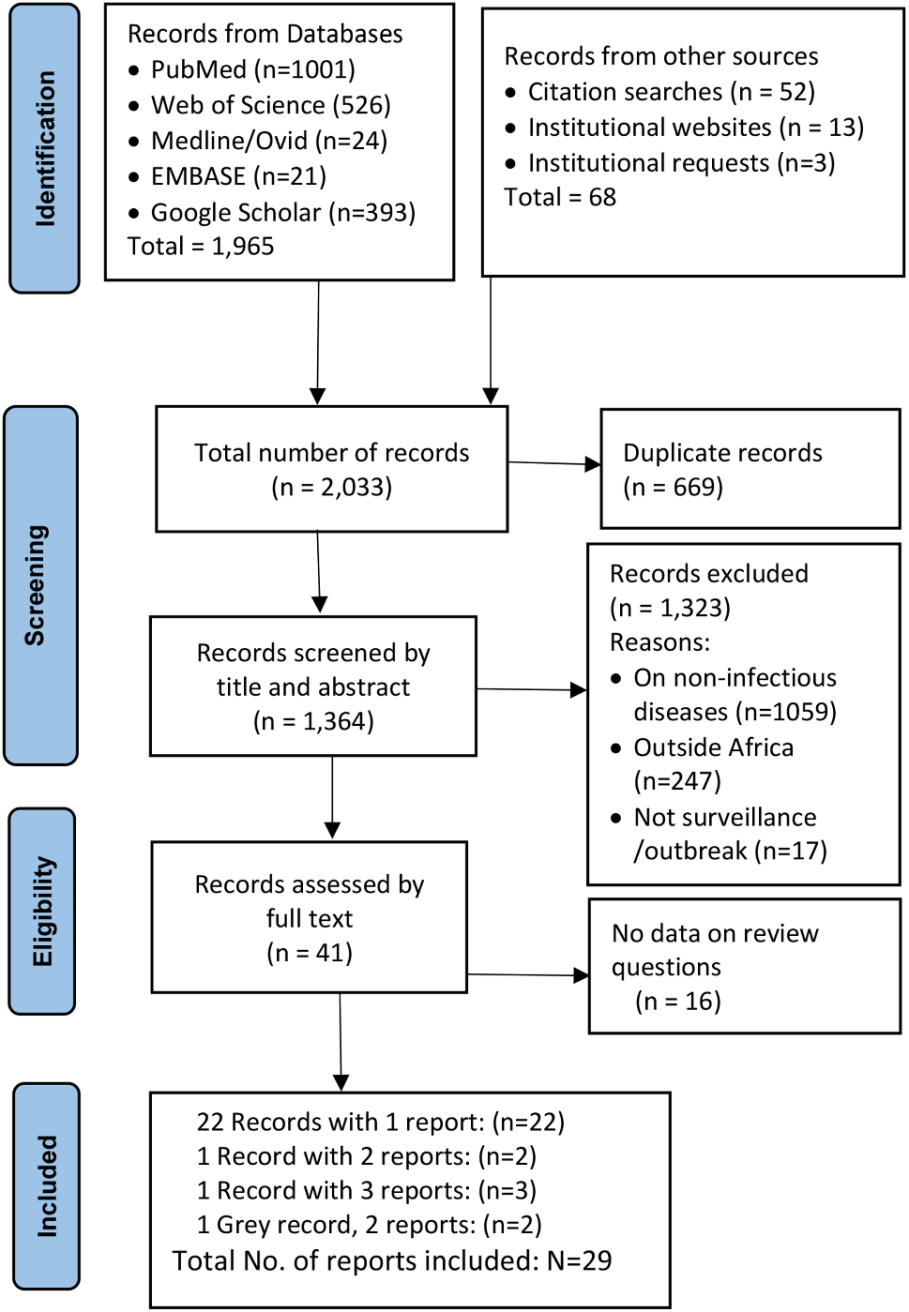
PRISMA flow diagram of record screening and selection process

All the searches yielded a total of 2,033 records, including 669 duplicates. After title and abstract screening of the remaining 1,364 unique records, we excluded 1,323 records based on the aforementioned criteria. Of the remaining 41 records, another 15 were excluded based on the full text assessment, leaving 25 records for inclusion. Owing to multiple case reporting in two of the included records, we obtained a total of 29 reports for analysis (Fig. 1).

### Study characteristics

The 29 reports described the implementation of a total of 27 projects at 32 implementation sites (Table 1). About 67% (18/27) of the projects used open-source tools. Four of the projects did not have specific names for their tools except general descriptive labels such as mHealth or web-based systems. The median number of reports published per year was 1 (range 0–4).

**Table 1:** General characteristics of reports on the implementation of digital tools for surveillance or outbreak response in Africa, 2003–2022

Author	Year publ.	Project Country	Disease focus	Name/Tool description	Source code	Devices used	Reporting mode
Muller et al., [38]	2009	Uganda	Malaria	HIMAL - EDS	Unspecified	Computers	Internet
	2009	Kenya	Malaria	HIMAL - EDS	Unspecified	Computers	Internet
Randrianasolo et al., [39]	2010	Madagascar	All notifiable diseases	No specific tool	Unspecified	Mobile phones	SMS
Asiimwe et al., [40]	2011	Uganda	Malaria	Rapid SMS	Open source	Mobile phones	SMS
Madder et al., [41]	2012	South Africa	Ticks and tick-borne diseases	SurveyToGo	Proprietory code	Computers Mobile Smartphones PDAs	Internet
	2012	Benin	Invasive tick species	EpiCollect	Open source	Mobile Smartphones	Internet
	2012	Kenya	Livestock diseases	EpiSurveyor & RapidSMS	Open source	Smartphones	Internet SMS
Rajatonirina et al., [42]	2012	Madagascar	Influenza-like illness	Web-based system	Unspecified	Mobile phones	SMS
Rajput et al., [43]	2012	Kenya	HIV/AIDS and TB	ODK collect with OpenMRS server	Open source	Smartphones Tablets	Internet
Chang et al., [29]	2013	Uganda	HIV/AIDS	No specific tool	Unspecified	Computers Mobile phones	SMS Internet
Githinji et al., [44]	2014	Kenya	Malaria	SMS for life	Unspecified	Computer Mobile phones	SMS
Mwabukusi et al., [45]	2014	Burundi, Tanzania & Zambia	Human & Animal diseases	EpiCollect and ODK app	Open source	Mobile Smartphones	Internet SMS
Mtema et al., [46]	2016	Tanzania	Human Rabies	OpenXdata	Open source	Mobile Smartphones Computers	GSM GPRS Internet
Karimuribo et al., [47]	2017	Tanzania	Human & Animal diseases	AfyaData	Open source	Smartphones	Internet
Kipanyula et al., [48]	2017	Tanzania	Human Rabies	Web GIS	Open source	Computers Smartphones	Internet
Oza et al., [49]	2017	Sierra Leone	Ebola Virus Disease	OpenMRS-Ebola^*^	Open source	Tablets Computers	Internet
Toda et al., [50]	2017	Kenya	One Health	mSOS	Unspecified	Mobile phones Smartphones	SMS
El-Khatib et al., [51]	2018	Ctr. African Rep.	All notifiable diseases	Argus	Unspecified	Smartphones Tablets Computers	SMS Internet
Maraba et al., [52]	2018	South Africa	Tuberculosis	mHealth**	Unspecified	Mobile phone Smartphones	SMS
Mohammed et al., [53]	2018	Ghana	Diseases-Children Under-5	eHiss	Unspecified	Mobile phones Smartphones	SMS
Coetzer et al., [54]	2019	Tanzania	Canine Rabies	DHIS2 coupled with RVT, RCS, REB, & GDL Manager	Open source	Smartphones Custom-made GDL devices	Internet
	2019	Zimbabwe	Canine Rabies	DHIS2 coupled with RVT, RCS, REB, & GDL Manager	Open source	Smartphone Custom-made GDL devices	Internet
Singh et al., [55]	2019	South Africa	HIV/AIDS	mHealth-system	Unspecified	Smartphones	SMS
Martin et al., [56]	2020	Sierra Leone	All notifiable diseases	eIDSR on DHIS2	Open source	Tablets Computers	Internet & SMS
Sloan et al., [57]	2020	Sierra Leone	All notifiable diseases	eIDSR on DHIS2	Open source	Tablets Computers	Internet
Moore et al. [58]	2021	Zambia	Malaria	mHAT web	Unspecified	Smartphones Tablets Computers	Internet
Njenga et al., [59]	2021	Kenya	Livestock diseases	KABS	Open source	Smartphones Tablets Computers	SMS
Grainger [60]	2020	Ghana	All notifiable diseases	SORMAS	Open source	Tablets Computers	Internet
	2020	Nigeria	All notifiable diseases	SORMAS	Open source	Tablets Computers	Internet
Turimumahoro et al., [61]	2022	Uganda	Tuberculosis	CommCare**	Open source	Tablets Computers	SMS

^&^Full meaning of all abbreviations on this table are available on list of abbreviations

^*^ Implemented for outbreak response in treatment center

^**^Implemented for case follow-up and/or contact investigation

### Quality of reports

Aside from the two grey reports, the rest of the reports (27) that were published in peer-reviewed journals presented their research objectives, methods, and results systematically. Of the 29 reports, 13 (45%) did not discuss the limitations of their studies, 10 (34%) did not include statements on competing or conflicts of interests, and 17 (59%) did not report on ethical approval (Appendix 2).

### Geographical reach of implementations

**Fig. 2:**
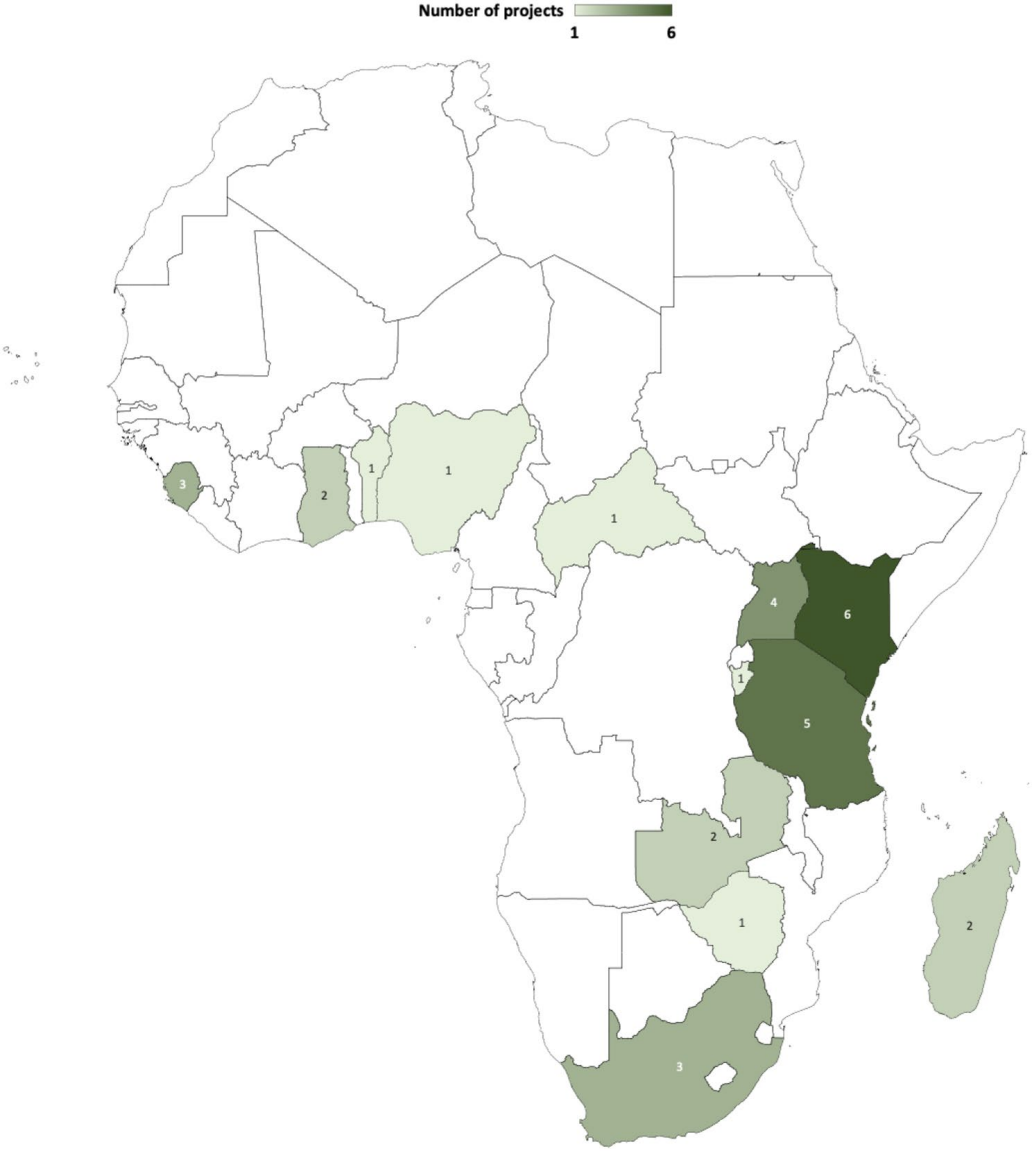
Distribution of number of projects reports by countries implementing digital tools for surveillance or outbreak response in Africa, 2003–2022

The 32 implementing sites were located across 13 countries with 24 (75%) from the eastern and southern Africa bloc. (Fig. 2). Three records reported on implementations across multiple countries (Table 1). One record reported malaria surveillance projects in Kenya and Uganda. A second reported three different but related case studies on the surveillance of tick and tick-borne disease in Benin, Kenya, and South Africa. A third reported a surveillance project on human and animal diseases in Burundi, Tanzania, and Zambia.

On the country level, the geographical reach of implementation at the 32 sites included 4 (13%) communities or cities, 17 (53%) district or sub-districts, 2 (6%) regions or provinces; and 9 (28%) with national coverage.

### Purpose and disease focus areas of implementations

**Fig. 3:**
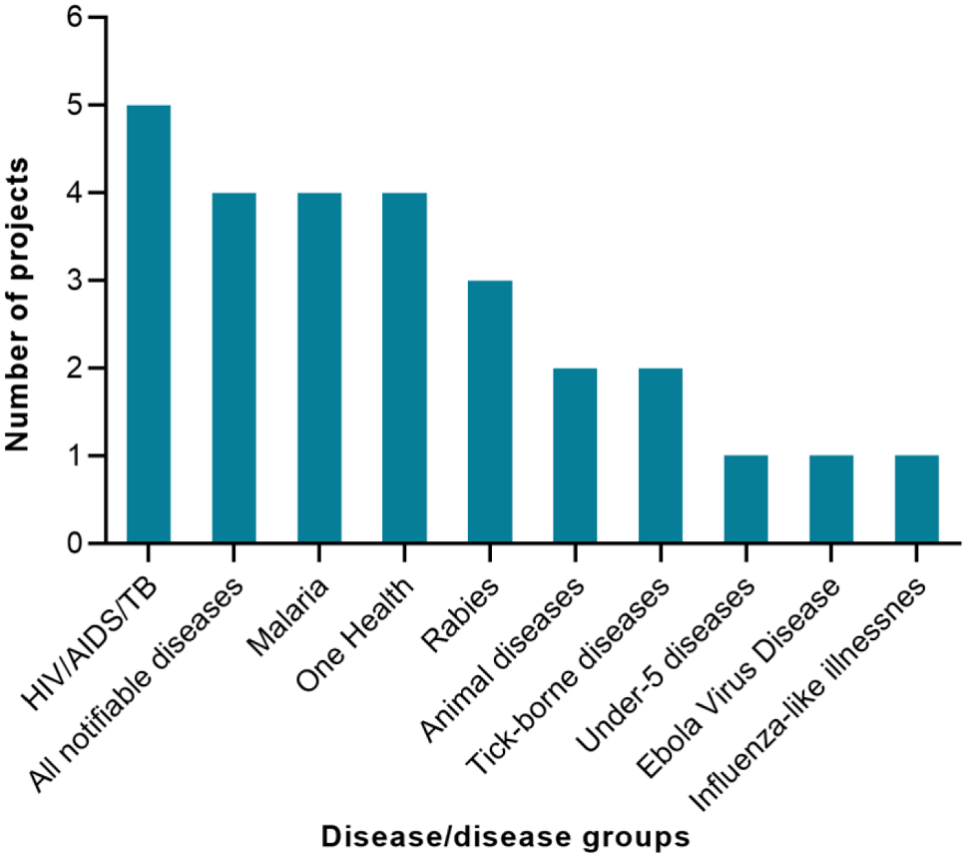
Diseases for which projects for digital surveillance or outbreak response were implemented in Africa, 2003–2022

Of the 27 projects, 21 (78%) were implemented mainly for surveillance, 5 (17%) for both surveillance and outbreak response, and 1 (3%) (OpenMRS-Ebola) for outbreak response. The implementations covered diseases of humans, animals, and One Health conditions (Fig. 3).

### Duration of implementations

**Fig. 4:**
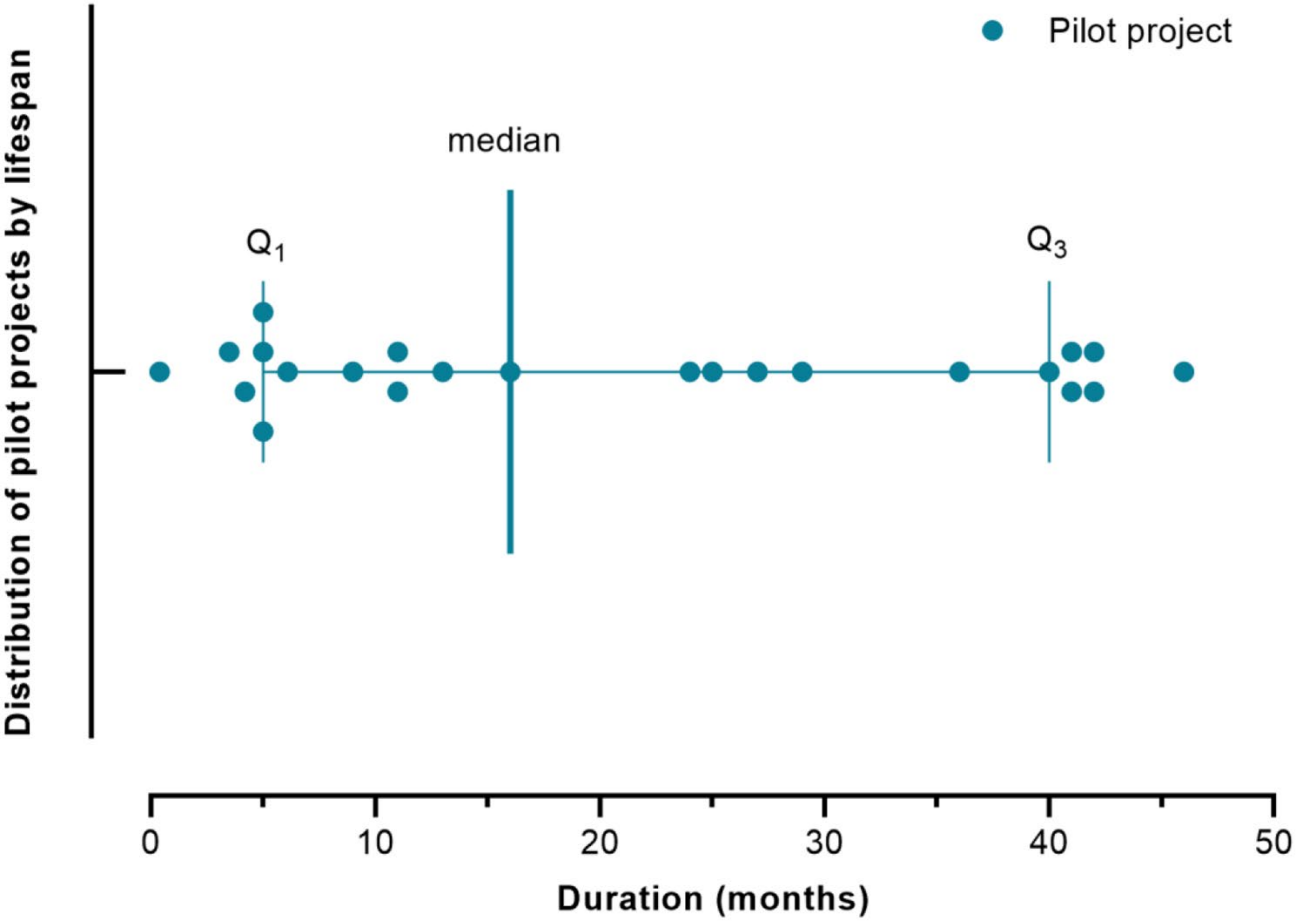
Duration of pilot implementations of digital projects for surveillance or outbreak response in Africa, 2003–2022

At the 32 implementation sites, 24 (75%) were pilot projects, 5 (16%) were ongoing, and 3 (9%) were one-time interventions. The durations of the pilot projects were reported for 23 of 24 implementation sites. The median duration of the pilot projects was 16 months, (IQR: 5–40) (Fig. 4). All the pilot projects reported some level of successful outcomes for which the implementers recommended scale ups whilst highlighting challenges to overcome.

### Reporting on cost components

**Fig. 5:**
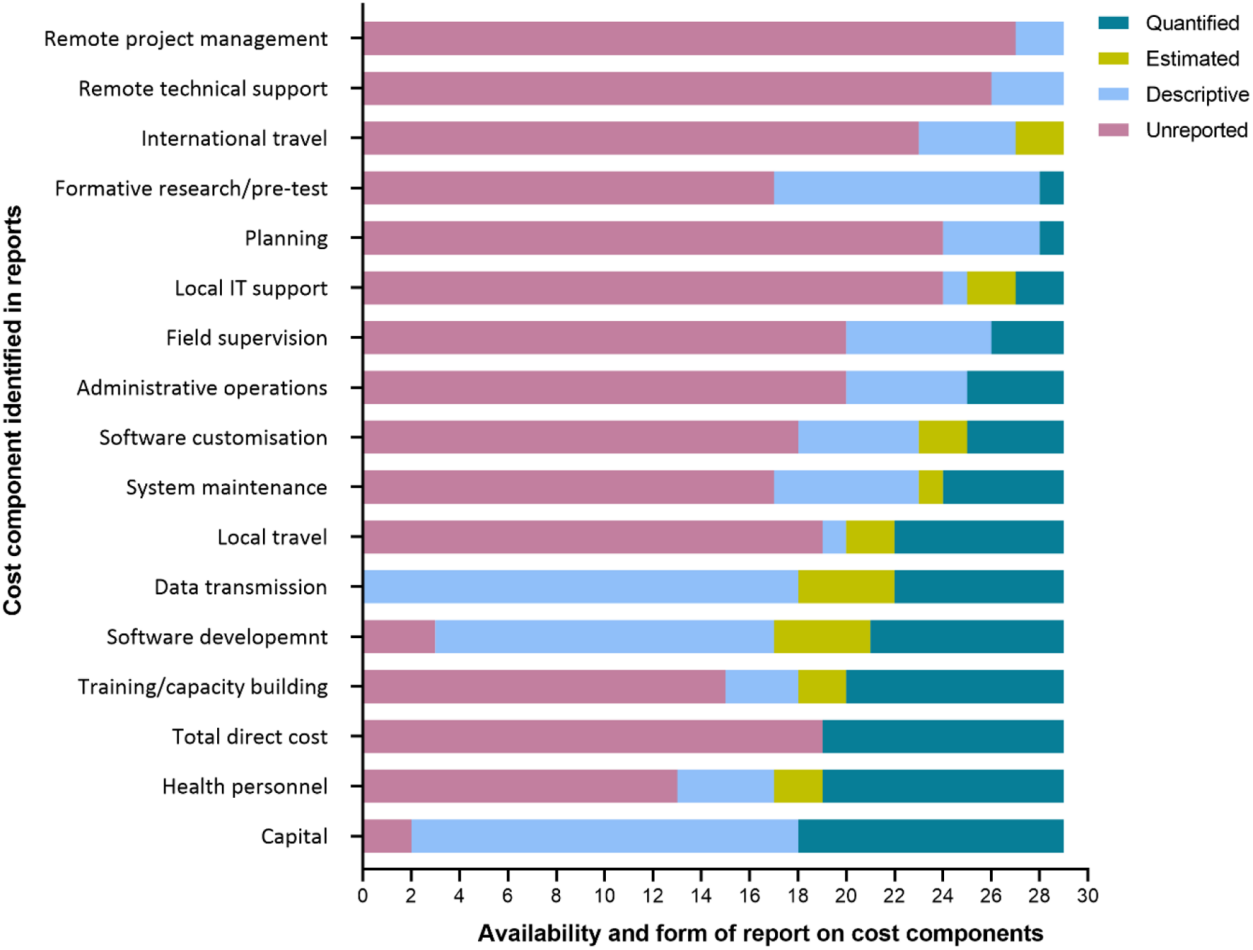
Reporting patterns for cost components in the implementation digital projects for surveillance or outbreak response in Africa, 2003–2022

The reporting of cost components for implementations varied in detail and approach based on the main objectives of each report. We identified a total of 17 cost components across all the reports (Fig. 5). On the more frequently quantified cost components across the 29 reports, 11 (38%) reported for capital costs (start-up infrastructure and pieces of equipment), 10 (34%) for health personnel compensation, 9 (31%) for training and capacity building, 8 (28%) for software maintenance, 7 (24%) for data transmission, and 7 (24%) for local travel. One report quantified the cost of planning. No report quantified the costs of international travel, remote technical support, and remote project management support of co-implementers in partner institutions outside Africa. The reporting of the costs of implementation varied in the range of activities undertaken. Some of the granular cost items included rent for office space, general office supplies, utility bills, cleaning services, and the monetary compensation of local health personnel in the forms of salaries, allowances, and per diems for meetings, trainings, and field supervisions. A set of the granular cost items pooled across the included reports under the cost components is summarised in Appendix 3.

Of the 29 reports, 10 (34%) provided information on the total direct costs of projects in monetary terms (USD) with varying combination of aggregate and cost breakdowns [29, 38, 40, 43, 46, 51, 57, 61]. The median total direct cost was 57,189.00 (the average of 50,036.88 for Rapid SMS system for malaria surveillance in two districts in 2009 for 11 months [40], and 64,342.00 for the pilot of eIDSR in one district for 14 weeks 2017 [57]). Overall, the total direct cost for these projects ranged from 2,353.27 USD (for an SMS and internet-based system implemented over 27 months from 2006 to 2008 in one district [29], to 472,327.00 USD (for the pilot of mHealth system over 41 months from 2014 to 2017 for a national capital city [61].

### External project funding support

A total of 39 main external funding institutions supported the implementation of 26 of the 27 projects. Of 65 counts of external funding sources, 35 (54%) were governmental agencies, 15 (23%) foundations, 7 (11%) UN agencies, 3 (5%) non-governmental organisations (NGOs), 3 (5%) industry, and 1 (2%) each for scientific societies, and the World Bank. (Table 2). The lead funders among governmental agencies based on the number of projects they supported were the US National Institute of Health (NIH), US Centers for Disease Control and Prevention (US-CDC), and US Agency for International Development (USAID). The lead funders among the foundations were the Bill and Melinda Gates Foundation, the Rockefeller Foundation, and the Wellcome Trust (Table 2). The WHO, UNICEF, FAO, and the World Bank were the global organisations which supported the implementation of digital disease surveillance or outbreak response systems. The three funding sources from industry were Google Inc., MSD (Merck, Sharp & Dohme) Animal Health, and Novartis Pharma AG. The funders mostly collaborated with local and external academic or research institutions, and the responsible government agencies or ministries of beneficiary countries.

**Table 2:** Types and sources of funding support for implementing digital tools for surveillance or outbreak response in Africa, 2003–2022

Funder	Type of funder	Source of funds	No. of projects
NIH	Government	USA	6
US CDC	Government	USA	6
WHO	Global organisation	UN	5
EU	Government	EU	3
FCDO	Government	UK	3
Rockefeller Foundation	Private	Foundation	3
USAID	Government	USA	3
Wellcome Trust	Private	Foundation	3
Bill and Melinda Gates Foundation	Private	Foundation	2
BMZ/GIZ	Government	Germany	2
US PEPFAR	Government	USA	2
BMBF	Government	Germany	1
FAO	Global organisation	UN	1
Google, Inc. (now Google LLC)	Private	Industry	1
National initiative	Government	Tanzania	1
Abbott Fund	Private	Foundation	1
AMED	Government	Japan	1
BELSPO	Government	Belgium	1
BMBF	Government	Germany	1
Burroughs Wellcome Fund	Private	Foundation	1
Compton Foundation	Private	Foundation	1
Doris Duke Charitable Foundation	Private	Foundation	1
IDRC	Government	Canada	1
IPM	Private	Scientific society	1
JICA	Government	Japan	1
MMV	Public Private Partnership	Foundation	1
MSD Animal Health	Private	Industry	1
MSF	Private	NGO	1
MSP	Government	France	1
NLM	Government	USA	1
Novartis Pharma AG	Private	Industry	1
Save the Children Fund	Private	NGO	1
Skoll Global Threats Fund	Private	Foundation	1
UBS Optimus Foundation	Private	Foundation	1
UNICEF	Global organisation	UN	1
US HHS	Government	USA	1
US PMI	Government	USA	1
WAP	Private	NGO	1
World Bank	Global organisation	World Bank	1

^&^Full meaning of all abbreviations on this table are available on list of abbreviations

## Discussion

Most of the implementations were on pilot basis,and hence limited in geographical reach. They covered single diseases or disease groups from the sets of notifiable diseases. The reports contained limited description and quantification of project cost components. The main sources of external funding support were intergovernmental co-operations and foundations. All the reports were from the sub-Saharan Africa region. This pattern of distribution of project reporting sites on the continent may be partly reflective of the relatively higher investment in the sub-Saharan Africa region in the last two decades. It could mirror a response to the increasing frequency of emerging and remerging infectious diseases of pandemic potential [62, 63]. The higher number of reports from the eastern and southern Africa bloc also reflects a relative higher number of projects arguably attributed to earlier mobile and internet penetration relative to other sub-Saharan Africa regions [64, 65].

The projects targeted both specific diseases such as malaria and rabies, as well as disease groups such as HIV/AIDs and tuberculosis, all notifiable diseases, and One Health events. Programmatic interventions for some of the traditional priority diseases such malaria, HIV/AIDs, and tuberculosis have long existed in Africa. However, the inclusion of digital surveillance for case detection, investigation, and follow ups have become add-ons for accelerating the achievement of disease prevention and control targets [66–68]. A case in point of coupling digital interventions to traditional disease control programmes is the WHO Global Taskforce on “Digital health in the TB response” [66]. We observed that about 30% of the reported implementations were either for all notifiable diseases or One Health conditions. This may be informed by the increasing recognition of the high burden of One Health conditions in the WHO-AFRO. It is reported by Talisuna et al. in their comprehensive review and mapping of the spatial and temporal distribution of infectious disease epidemics, disasters and other potential public health emergencies in the region [69].

Most of the implementations were limited in geographical reach and duration because they were pilot projects. This finding is consistent with the widespread phenomenon where many technical projects are not scaled even after successful piloting, especially in low-and middle-income countries (LMICs) [70–73]. In an effort to regulate and promote implementation of eHealth initiatives at scale in Uganda, the director general of health services issued a moratorium in 2012 directing the immediate halting of all projects [74]. The moratorium cited among other reasons, the need for actors to demonstrate compliance with national requirements, present convincing mechanisms of sustainability, and provide clarity on system ownership. However, Gimbel et al. suggested that the heavy reliance of LMICs on external donors, governments, and private sector for funding partly explains the increasing trend of pilot projects that do not get scaled up [68].

Most of the reports identified by the present review did not include cost reporting as an outcome. Similar to the majority of reports on implementation outcomes, the literature reporting implementations of digital projects are limited on cost information compared to other outcomes such as acceptability, appropriateness, and feasibility [75–77]. Even though a scoping review by Silenou et al. reported the use of digital applications from 28 African countries as of 2021 [6], we obtained eligible reports with information on cost considerations from only 13 countries. About 28% of our included reports provided some form of quantitative cost information. On face value, this finding is less grim given that in their systematic review including 235 implementation studies, Eisman et al. reported that only about 10% of these provided cost information [78].

The reports included in our review provide varying details of cost information on capital investments, data transmission, training, and software development. Even though the cost information was mostly descriptive, some reported cost analyses of implementation expenditures in various categorisations and detail [29, 38, 40, 43, 46, 51, 57, 61]. All the 10 reports that provided total cost of implementations were pilots, limited in geographical scale (districts, sub-districts, and communities), and disease-specific except one [57]. In addition, these projects varied widely in time horizon of initiation, duration, and country of implementation. This heterogeneity and the limited reporting of unit cost data have not allowed for meta-analysis. Still, these total costs may be useful for providing historical and situational context. This is especially true for actors who may consider similar projects in future beyond piloting to sustainable implementation at scale. In our experience as co-implementers of SORMAS in Ghana and Nigeria, we note that the total costs of implementation are underestimated. The reason being that many hidden and indirect costs are not accounted for. Further, even the total direct costs are difficult to track and compute because of the multiplicity of both internal and external contributing sources at various administrative levels of the health system. In the few cases where the quantification of cost information is reported, even fewer provide cost breakdowns in the main text, or as supplementary material [38, 40, 46, 57, 61]. This trend of aggregate reporting limits the possibilities for comprehensive economic evaluations of implementations. Shield et al. reported on this in their paper on factors limiting cost-effectiveness analysis [79], and Fukuda and Imanaka reported the same challenge in their assessment of the transparency of cost estimates in economic evaluations of patient safety programmes [80]. Thus, the incomplete reporting and quantification of cost components limit the availability of the needed raw material for sophisticated but useful health economics research, as well as evidence synthesis. Ultimately, this phenomenon deprives global health actors of guidance on context-relevant evidenced-based implementation strategies.

Aside the aforementioned difficulty in determining indirect costs, some fairly direct and tangible costs are simply not reported. This does not only compound the challenge of cost underestimation and limitations for evidence generation and synthesis;  it also limits efforts at estimating returns on investments and evaluating business models for implementations. For example, nearly all the implementations in our review benefited from participation of external co-implementers, but the costs of international travel, remote technical assistance and project management support were mostly unreported. Given that most of the implementations were pilots, these cost components could constitute a significant start-up cost – a well-recognised early barrier to implementation [77]. This pattern of reporting is consistent across most implementation studies. Aggregate reports on broad categories of cost components such as capital investments, personnel, and transport are more common compared to granular activity-based expenditures [76, 81, 82]. This trend of limited reporting of implementation costs may suggest a poor culture of systematic documentation, or open communication. The bottom line is that this challenge does not allow for comprehensive evaluation of return on investments. In turn, it hinders justification for more investments from funders [78, 83]. The limited reporting on cost may be attributed to organisational practices on financial confidentiality. However, Cidav et al. suggest that the lack of clearly defined and standardised costing methods for planning, execution, and evaluation of implementations could partly explain this phenomenon [76]. Hence, they propose the application of what they describe as “a pragmatic method for costing implementation strategies using time-driven activity-based costing” in conjunction with Proctor et al.’s framework on specifying and reporting implementation strategies [76, 84].

We identified a wide base of external funding support for the implementations. However, about 75% of the projects were funded through grants. The grant durations were shorter than four years, half of them lasting 16 months or shorter. The main sources of external funding support were governmental agencies and foundations. We observed that the implementations were fragmented in purpose within countries and among funders. The open sharing of cost information promotes transparency, donor confidence, and a better contextualisation of implementation demands [85–87]. On the contrary, the lack of coordination coupled with limited open reporting on initiatives contributes to duplications and detracts from incremental progress in overall health system strengthening in Africa [88, 89]. In their systematic review on the politics of disease control in Africa, Chattu et al. reiterated how fragmented funding of disease-specific interventions inadvertently undermine health system strengthening in developing countries [89, 90]. That said, deployments of digital outbreak response systems such as the OpenMRS-Ebola in Sierra Leone as a one-time intervention [49] remain critical strategies for enhancing emergency response to major outbreaks. In this regard, funding mechanisms and reporting practices of such interventions depend more on the evolution of the emergency, and less on long-term implementation strategies. Still, the latter would be expected for the digitalisation of national surveillance systems through for example DHIS2 or SORMAS. For the foreseeable future we expect funding mechanisms and reporting requirements to vary depending on the funder and the purpose of the system. However, to the extent that external funding support is what it is – *support*, the onus of achieving sustainable implementation is on the beneficiary countries. For example, some of the reports we analysed captured recurrent expenditures such as rent for office space, utility bills, and extra compensation for already employed local health personnel [38, 42, 46, 61]. This suggests that in some cases, there is little or no collaboration between external implementing actors and the relevant state agencies. Aside missing the opportunity of saving on some bills, the lack of close collaborations also detracts from the prospects of skill and technology transfer to local personnel.

Even though we do not find literature that focused on the availability of published evidence for digitalisation of surveillance and outbreak response, the findings on limited transparency in cost reporting may resonate to various extents in other continents. For example, in their article on the dawn of digital public health in Europe [91], the representatives of the European Public Health Association’s digital health section underscored how the exigencies of the COVID-19 pandemic response have shifted stakeholder perceptions of digital surveillance tools from one of “opportunities” to “necessities”. Thus, before then, we may infer with caution, that the national and regional actors were less likely instituted deliberate funding and governance procedures beyond the routine institutional requirements to address any limitations in documentation and public reporting of expenditures. It may still be the case. Similar systematic reviews of the current practices on this subject in other continents could offer more insights and allow for comparisons of prevailing practices on evidence sharing so to exchange lessons.

In sum, our review substantiates the fragmentation of digitalisation efforts in Africa in the last 20 years; highlights the prevailing culture on open reporting of implementation cost data; and underscores the urgency for broad multi-level stakeholder engagements and resource commitments for operating comprehensive digital systems at scale in the ultimate interest of global health. To address the challenge of fragmentation, funding institutions should consider conducting joint reviews and approaches which may align with their vision and mandate so to minimise the risks of duplication. Such a review could reveal possibilities for synergy, encourage collaborations, benefit from joint governance and transparent reporting, and consolidate the gains of earlier projects. Ultimately, it could increase the scale and sustainability of implementations. For improving the documentation and transparent cost reporting of future projects, we recommend that as part of routine project reporting, a granular cost reporting template be included as a mandatory appendix for reporting implementation cost breakdowns to funding agencies. Where applicable, further funding release should be contingent on a positive evaluation of cost reporting and transparency. Further, our findings hold some implications for improving practices on the funding and implementation of digital systems for public health surveillance in Africa and comparable settings. First, our review demonstrate that actors cannot rely on openly published grey and peer reviewed literature for evidence on cost of digitalisation of surveillance in Africa. Second, regarding the high failure rate of projects, our findings suggest that tying external funding support to commitments of national actors for system ownership could deliver projects with improved sustainability potential. Third, by highlighting the limited reporting of evidence on implementation costs, our findings seek to underscore lost opportunities for improving cost planning and forecasting. . Our findings also beg the question of what underlying factors could explain the limited sharing of experiences on implementation costs. This question could be tabled for panel discussions at workshops and conferences on implementation research in digital health and related topics.

For future research, we recommend an extensive review of unpublished institutional financial reports, complemented with multidisciplinary expert inputs using the Delphi approach [92]. This would allow stakeholders to obtain a comprehensive set of cost components, as well as historical and prevailing cost estimates. These could form a basis for developing a living cost estimating matrix to guide financial planning, forecasting, and cost reporting for the implementation of digital systems for infectious disease surveillance or outbreak response in different settings. A multi-country qualitative study would also be useful in unravelling contextual and systemic factors that limit access to cost data for implementation research in general. The reporting on data ownership, confidentiality, and security of the systems we reviewed was not within the scope of our study. Nevertheless, we are convinced transparent reporting on project implementation that includes these important ethical dimensions would increase clarity of responsibilities for system governance and promote sustainability.

### Limitations

The risk of publication bias is the main limitation of our review. The reason is that project implementation information that is openly available is hardly complete compared to the unpublished reports. To minimise this bias, we communicated with some authors and institutions to request additional information in keeping with our review questions. Also, where the reporting of a cost component was not directly spelt out in reports but could be reasonably inferred, there was the risk of misclassification of reporting. To minimise this, we relied on discussion among at least three authors to reach a consensus.

## Conclusions

The evidence on costing data for the digitalisation of surveillance and outbreak response in the published literature is sparse in quantity, limited in detail, and without a standardised reporting format. This detracts from incremental learning from past funding pitfalls that would otherwise improve funding strategies for future projects. Most initial direct project costs are substantially donor dependent, short lived, and thus unsustainable. National public health institutions in Africa and donor partners should consider promoting standardisation and open reporting of implementation cost data to inform better design and planning of future digitalisation projects. In keeping with their mandate under the international health regulations, African governments should commit financially to the long-term sustainability of digital surveillance systems. Supporting donor partners should also endeavour to engage beyond piloting.

### Electronic supplementary material

Below is the link to the electronic supplementary material.

Supplementary Material 1

Supplementary Material 2

Supplementary Material 3

## Data Availability

All data generated or analysed during this study are included in this published article and its supplementary information files.

## Abbreviations

DHIS2: District Health Information System ? version 2
HIMAL EDS: Highland Malaria Project Early Detection System (for epidemic malaria)
mSOS: Mobile SMS-based disease outbreak alert system
RCS: Rabies Case Surveillance
AMED: Japan Agency for Medical Research and Development
BELSPO: Belgian Science Policy Office
BMBF: Federal Ministry of Education and Research (Bundesministerium f?r Bildung und Forschung)
eIDSR: Electronic Integrated Disease Surveillance and Response
EU: European Union
FCDO: Foreign, Commonwealth and Development Office
GIZ: GARC (Global Alliance for Rabies Control) Data Logger
IDRC: German Corporation for International Cooperation (Gesellschaft f?r Internationale Zusammenarbeit)
IPM: International Development Research Centre of Canada
IPM: Pasteur Institute of Madagascar (Institut Pasteur de Madagascar)
JICA: The Japan International Cooperation Agency
KABS app: Kenya Animal Biosurveillance System application
mHAT app: Mobile Health and Treat application
MMV: Medicines for Malaria Ventures
MSD Animal Health: Merck, Sharp and Dohme Corp Animal Health
MSF: Doctors Without Borders (Medecins Sans Frontieres)
MSP: Ministry of Health Prevention (Ministere de la Sante et de la Prevention)
NIH: National Institutes of Health
NLM: National Library of Medicine
OpenMRS: Open Data Kit
Ebola app: Open Medical Records System - Ebola application
Rapid SMS: Rapid Short Messaging Service
REB: Rabies Epidemiological Bulletin
RVT: Rabies Vaccine Tracker
SORMAS: Surveillance, Outbreak Response Management and Analysis Systems
STG: SurveyToGo
UBS Optimus Foundation: Union Bank of Switzerland Optimus Foundation
UNICEF: United Nations Children’s Fund (originally: United Nations International Children’s Emergency Fund)
US CDC: United States Centers for Disease Control and Prevention
US HHS: United States Health and Human Services
US PEPFAR: United States President’s Emergency Plan for AIDS Relief
US PMI: United States President’s Malaria Initiative
USAID: United States Agency for International Development
WAP: World Animal Protection (formerly The World Society for the Protection of Animals - WSPA)
Web GIS: Web-based Geographic Information System
WHO: World Health Organisation
WAP: World Animal Protection (formerly The World Society for the Protection of Animals - WSPA)
