# Supplementary material for: Availability of published evidence on coverage, cost components, and funding support for digitalisation of infectious disease surveillance in Africa, 2003–2022: a systematic review
Source: BMC Public Health. 2024 Jun 28;24:1731. doi: 10.1186/s12889-024-19205-2 (PMC11214246; doi:10.1186/s12889-024-19205-2)
Supplement: Supplementary file 1 — Supplementary Material 1 [file 12889_2024_19205_MOESM1_ESM.docx]

**Appendix 1: Search strategies for different databases**

1. **PubMed**

***Search Terms***

***Search # 1: cost or cost components***

*Cost* OR cost component*OR cost element* OR cost analysis* OR cost evaluation* OR evaluat* cost OR cost effective* OR implementat* cost* OR project cost* OR program* cost* OR cost calcuati* OR fund* OR financ* OR capital cost OR training cost OR supervision cost OR personnel cost OR operational cost OR maintenance cost*

***Search #2: digital health tools***

*digital tool* OR eHealth OR e-Health OR mHealth OR Mobile Health OR contact tracing tool* OR contact follow-up tool* OR contact tracing App* OR contact follow-up App* OR contact monitoring tool* OR Realtime case monitoring tool* OR mobile phone* as surveillance tool* OR android$based mhealth OR android$based mobile health OR android$based e$health*

***Search #3: Disease surveillance or outbreak response***

*eSurveillance OR electronic surveillance OR digital surveillance OR disease surveillance OR Infectious disease surveillance OR public health surveillance OR Surveillance OR outbreak* OR epidemic* OR pandemic* OR outbreak respon* OR epidemic respon* OR pandemic respon* OR outbreak control* OR epidemic control* OR pandemic control* OR outbreak manag* OR epidemic manag* OR pandemic manag* OR epidemic preparedness OR pandemic preparedness OR outbreak preparedness OR public health emergenc**

***Search #4: Setting of review***

*Afric* OR Sub-Sahara* Afric* OR West Afric* OR East Afric* OR North Afric* OR South* Afric* OR Central Afric* OR developing countr* OR Low and middle income countr* OR LMIC* OR resource limited countr* OR resource poor countr* OR Resource limited setting* OR Resource poor setting* OR resource$constrained setting* OR low-income setting* OR low income countr* OR middle-income countr**

***Complete Search (# 5): #1 AND #2 AND #3 AND #4***

1. **Medline over Ovid**

***Search # 1:*** ***cost OR cost components***

*Cost* OR cost component*OR cost element* OR cost analysis* OR cost evaluation* OR evaluat* cost OR cost effective* OR implementat* cost* OR project cost* OR program* cost* OR cost calcuati* OR fund* OR financ**

***Search #2: digital tools for health***

*digital tool* OR eHealth OR e-Health OR mHealth OR Mobile Health OR contact tracing tool* OR contact follow-up tool* OR contact tracing App**

***Search #3: Disease surveillance and outbreak response***

*eSurveillance OR electronic surveillance OR digital surveillance OR disease surveillance OR Infectious disease surveillance OR public health surveillance OR Surveillance OR outbreak* OR epidemic* OR pandemic* OR outbreak respon* OR epidemic respon**

***Search #4: Setting of review***

*Afric* OR Sub-Sahara* Afric* OR West Afric* OR East Afric* OR North Afric* OR South* Afric* OR Central Afric* OR developing countr* OR Low and middle income countr* OR LMIC* OR resource limited countr**

***Complete Search (# 5): #1 AND #2 AND #3 AND #4***

1. **Web of Science**

*(((ALL=(Cost* OR cost component*OR cost element* OR cost analysis* OR cost evaluation* OR evaluat* cost OR cost effective* OR implementat* cost* OR project cost* OR program* cost* OR cost calcuati* OR fund* OR financ*)) AND ALL=(digital tool* OR eHealth OR e-Health OR mHealth OR Mobile Health OR contact tracing tool* OR contact follow-up tool* OR contact tracing App*)) AND ALL=(eSurveillance OR electronic surveillance OR digital surveillance OR disease surveillance OR Infectious disease surveillance OR public health surveillance OR Surveillance OR outbreak* OR epidemic* OR pandemic* OR outbreak respon* OR epidemic respon*)) AND ALL= (Afric* OR Sub-Sahara* Afric* OR West Afric* OR East Afric* OR North Afric* OR South* Afric* OR Central Afric* OR developing countr* OR Low and middle income countr* OR LMIC* OR resource limited countr*)*

1. **Embase**

***#1*** *((((((((((cost* OR 'cost'/exp OR cost) AND component* OR 'cost'/exp OR cost) AND element* OR 'cost'/exp OR cost) AND analysis* OR 'cost'/exp OR cost) AND evaluation* OR 'evaluat* cost' OR (evaluat* AND ('cost'/exp OR cost)) OR 'cost'/exp OR cost) AND effective* OR implementat*) AND cost* OR project) AND cost* OR program*) AND cost* OR 'cost'/exp OR cost) AND calcuati* OR fund* OR financ* OR 'capital cost' OR (('capital'/exp OR capital) AND ('cost'/exp OR cost)) OR 'training cost' OR (('training'/exp OR training) AND ('cost'/exp OR cost)) OR 'supervision cost' OR (('supervision'/exp OR supervision) AND ('cost'/exp OR cost)) OR 'personnel cost' OR (('personnel'/exp OR personnel) AND ('cost'/exp OR cost)) OR 'operational cost' OR (operational AND ('cost'/exp OR cost)) OR 'maintenance cost' OR (('maintenance'/exp OR maintenance) AND ('cost'/exp OR cost))) AND [2003-2022]/py*

***#2*** *(digital AND tool* OR 'ehealth'/exp OR ehealth OR 'e health'/exp OR 'e health' OR 'mhealth'/exp OR mhealth OR 'mobile health'/exp OR 'mobile health' OR (mobile AND ('health'/exp OR health)) OR (('contact'/exp OR contact) AND tracing AND tool*) OR (('contact'/exp OR contact) AND ('follow up'/exp OR 'follow up') AND tool*) OR 'contact tracing'/exp OR 'contact tracing' OR (('contact'/exp OR contact) AND tracing AND app*) OR 'contact follow-up' OR (('contact'/exp OR contact) AND ('follow up'/exp OR 'follow up') AND app*) OR 'contact monitoring' OR (('contact'/exp OR contact) AND ('monitoring'/exp OR monitoring) AND tool*) OR 'realtime case monitoring' OR (('realtime'/exp OR realtime) AND case AND ('monitoring'/exp OR monitoring) AND tool*) OR 'mobile as surveillance' OR (mobile AND phone* AND as AND ('surveillance'/exp OR surveillance) AND tool*) OR 'android$based mhealth' OR (android$based AND ('mhealth'/exp OR mhealth)) OR 'android$based mobile health' OR (android$based AND mobile AND ('health'/exp OR health)) OR android$based) AND e$health AND [2003-2022]/py*

***#3*** *((((((((((esurveillance OR 'electronic surveillance' OR (electronic AND ('surveillance'/exp OR surveillance)) OR 'digital surveillance' OR (digital AND ('surveillance'/exp OR surveillance)) OR 'disease surveillance'/exp OR 'disease surveillance' OR (('disease'/exp OR disease) AND ('surveillance'/exp OR surveillance)) OR 'infectious disease surveillance' OR (infectious AND ('disease'/exp OR disease) AND ('surveillance'/exp OR surveillance)) OR 'public health surveillance'/exp OR 'public health surveillance' OR (('public'/exp OR public) AND ('health'/exp OR health) AND ('surveillance'/exp OR surveillance)) OR 'surveillance'/exp OR surveillance OR outbreak* OR epidemic* OR pandemic* OR 'outbreak'/exp OR outbreak) AND respon* OR 'epidemic'/exp OR epidemic) AND respon* OR 'pandemic'/exp OR pandemic) AND respon* OR 'outbreak'/exp OR outbreak) AND control* OR 'epidemic'/exp OR epidemic) AND control* OR 'pandemic'/exp OR pandemic) AND control* OR 'outbreak'/exp OR outbreak) AND manag* OR 'epidemic'/exp OR epidemic) AND manag* OR 'pandemic'/exp OR pandemic) AND manag* OR 'epidemic preparedness' OR (('epidemic'/exp OR epidemic) AND ('preparedness'/exp OR preparedness)) OR 'pandemic preparedness' OR (('pandemic'/exp OR pandemic) AND ('preparedness'/exp OR preparedness)) OR 'outbreak preparedness' OR (('outbreak'/exp OR outbreak) AND ('preparedness'/exp OR preparedness)) OR 'public health'/exp OR 'public health' OR (('public'/exp OR public) AND ('health'/exp OR health) AND emergenc*)) AND [2003-2022]/py*

***#4*** *(((((((((((afric* OR 'sub sahara*') AND afric* OR west) AND afric* OR east) AND afric* OR north) AND afric* OR south*) AND afric* OR 'central'/exp OR central) AND afric* OR developing) AND countr* OR low) AND ('middle income'/exp OR 'middle income' OR (middle AND ('income'/exp OR income) AND countr*)) OR lmic* OR (resource AND limited AND countr*) OR (resource AND poor AND countr*) OR 'resource limited' OR (resource AND limited AND setting*) OR 'resource poor' OR (resource AND poor AND setting*) OR resource$constrained) AND setting* OR 'low income'/exp OR 'low income') AND setting* OR 'low income'/exp OR 'low income' OR (low AND ('income'/exp OR income) AND countr*) OR 'middle income'/exp OR 'middle income') AND countr* AND [2003-2022]/py*

***#5: Complete Search: #1 AND #2 AND #3 AND #4***

1. **Google Scholar Advance Search:**

***Key words:***

*Cost, cost components, cost analysis, implementation, digital tools, mHealth, SMS-based surveillance, outbreak response infectious diseases Africa*

*Filters: All fields*

*Date: 2003 -2022*
