# Supplementary material for: Availability of published evidence on coverage, cost components, and funding support for digitalisation of infectious disease surveillance in Africa, 2003–2022: a systematic review
Source: BMC Public Health. 2024 Jun 28;24:1731. doi: 10.1186/s12889-024-19205-2 (PMC11214246; doi:10.1186/s12889-024-19205-2)
Supplement: Supplementary file 3 — Supplementary Material 3 [file 12889_2024_19205_MOESM3_ESM.docx]

**Appendix 3:** Granular implementation cost items from included reports (N=29)

| **Cost Component** | **Breakdown** |
| --- | --- |
| **Capital/Startup cost** | Building infrastructure |
|  | Vehicles |
|  | Servers |
|  | Tablets |
|  | Protective cases |
|  | Desktop computers |
|  | Laptop computers |
|  | Laptop bags |
|  | Mobile phones |
|  | PDA |
|  | SIM cards |
|  | Internet modems |
|  | Charger adopters |
|  | Car chargers |
|  | AC chargers/Converters |
|  | Printers |
|  | Photocopiers |
|  | Lead-Acid batteries power backup |
|  | Solar power backup system |
|  | PowerGorilla backup system |
| **Planning** | Site visits |
|  | Stakeholder engagement meetings |
|  | Virtual meetings |
|  | Telophone discussions |
| **Software developemnt** | Design thinking workshops |
|  | Feature specification |
|  | Release testing |
|  | Field testing |
| **Software customisation** | Design thinking workshops/meetings |
|  | Feature adaption |
|  | Release testing |
|  | Field testing |
| **Training** | Training materials (eg.brochure design & printing) |
|  | Catering |
|  | Per diem |
|  | Accommodation |
|  | Rentals (training venue) |
| **Field supervision** | Lodging |
|  | Per diems |
| **Formative Res./Pre-test** | Study logistics |
|  | Researcher allowances |
|  | Study scholarships |
|  | Participation incentive |
| **Health personnel** | Salaries |
|  | Allowances |
|  | Benefits |
|  | Incentives |
| **Admin. operations** | Office security |
|  | Electricity |
|  | Water |
|  | Internet data bundles subscriptions |
|  | Airtime/SMS credits subscriptions |
|  | General waste removal |
|  | Cleaning services |
|  | Office supplies (eg. printing, lamination, binding) |
|  | Management software |
|  | Overhead costs |
|  | Transaction taxes |
| **Data transmission (users)** | Internet data bundles subscriptions |
|  | Airtime/SMS credits subscriptions |
| **Local travel** | Vehicle rentals |
|  | Fuel (owned vehicle) |
|  | Vehicle maintenance |
|  | Per diems |
| **Local IT support** | Consultancy fees |
|  | Salaries (full/part-time services) |
|  | Data hosting fees |
| **System maintenance** | Annual software subcrition fees (e.g. CommCare) |
|  | Operating cost |
|  | Device replacement |
|  | Device repairs |
|  | Accessory replacement |
|  | Cyber security |
| **International travel** | Flight tickets |
|  | Travel insurrances |
|  | Visa fees |
|  | Lodging |
|  | Per diems |
| **Remote project management** | Salaries/compensations |
|  | Communications |
|  | Stationery and office supplies |
| **Remote technical support** | Programming |
|  | Debugging |
|  | Software curation |
